# Discovering Cohorts of Pregnant Women From Social Media for Safety Surveillance and Analysis

**DOI:** 10.2196/jmir.8164

**Published:** 2017-10-30

**Authors:** Abeed Sarker, Pramod Chandrashekar, Arjun Magge, Haitao Cai, Ari Klein, Graciela Gonzalez

**Affiliations:** ^1^ Department of Biostatistics, Epidemiology and Informatics Perelman School of Medicine University of Pennsylvania Philadelphia, PA United States; ^2^ Department of Biomedical Informatics Arizona State University Scottsdale, AZ United States

**Keywords:** natural language processing, machine learning, text mining, social media, pregnancy, cohort studies, data analysis

## Abstract

**Background:**

Pregnancy exposure registries are the primary sources of information about the safety of maternal usage of medications during pregnancy. Such registries enroll pregnant women in a voluntary fashion early on in pregnancy and follow them until the end of pregnancy or longer to systematically collect information regarding specific pregnancy outcomes. Although the model of pregnancy registries has distinct advantages over other study designs, they are faced with numerous challenges and limitations such as low enrollment rate, high cost, and selection bias.

**Objective:**

The primary objectives of this study were to systematically assess whether social media (Twitter) can be used to discover cohorts of pregnant women and to develop and deploy a natural language processing and machine learning pipeline for the automatic collection of cohort information. In addition, we also attempted to ascertain, in a preliminary fashion, what types of longitudinal information may potentially be mined from the collected cohort information.

**Methods:**

Our discovery of pregnant women relies on detecting pregnancy-indicating tweets (PITs), which are statements posted by pregnant women regarding their pregnancies. We used a set of 14 patterns to first detect potential PITs. We manually annotated a sample of 14,156 of the retrieved user posts to distinguish real PITs from false positives and trained a supervised classification system to detect real PITs. We optimized the classification system via cross validation, with features and settings targeted toward optimizing precision for the positive class. For users identified to be posting real PITs via automatic classification, our pipeline collected all their available past and future posts from which other information (eg, medication usage and fetal outcomes) may be mined.

**Results:**

Our rule-based PIT detection approach retrieved over 200,000 posts over a period of 18 months. Manual annotation agreement for three annotators was very high at kappa (κ)=.79. On a blind test set, the implemented classifier obtained an overall F_1_ score of 0.84 (0.88 for the pregnancy class and 0.68 for the nonpregnancy class). Precision for the pregnancy class was 0.93, and recall was 0.84. Feature analysis showed that the combination of dense and sparse vectors for classification achieved optimal performance. Employing the trained classifier resulted in the identification of 71,954 users from the collected posts. Over 250 million posts were retrieved for these users, which provided a multitude of longitudinal information about them.

**Conclusions:**

Social media sources such as Twitter can be used to identify large cohorts of pregnant women and to gather longitudinal information via automated processing of their postings. Considering the many drawbacks and limitations of pregnancy registries, social media mining may provide beneficial complementary information. Although the cohort sizes identified over social media are large, future research will have to assess the completeness of the information available through them.

## Introduction

### Pregnancy Exposure Registries

Premarket clinical trials assess the safety of medications in limited settings, and so, the effects of those medications on particular cohorts (eg, pregnant women, children, or people suffering from specific conditions) cannot be assessed. Pregnant women are actively excluded from clinical trials during the development of new medications because of fetal safety concerns [1]. Therefore, once a medication is released into the market, there is typically no data available to assess the fetal effects of in utero exposure other than from animal reproductive toxicology studies [2]. However, conclusions derived from animal studies may not generalize to humans [3]. Spontaneous reporting systems, such as the Food and Drug Administration Adverse Event Reporting System, are used for postmarketing drug safety surveillance, and they provide a mechanism for reporting adverse events associated with medication consumption. Although these sources may accumulate medication safety knowledge about specific population groups, studies have shown that they suffer from various problems such as underreporting, lack of denominator data, and absence of controls [2,4]. In addition, postmarketing surveillance techniques such as spontaneous reporting systems are retrospective in nature, with cases enrolled based on adverse outcome reporting from an unknown number of exposed pregnancies, making the samples biased toward adverse outcomes.

To address these issues, pregnancy exposure registries are developed for new medications. These registries enroll women prospectively (eg, after exposure but before childbirth) in a voluntary fashion and follow them for the entire duration of the pregnancy or longer. This design of pregnancy exposure registries enables researchers to conduct prospective observational studies, which are superior to retrospective studies because of the biases associated with the latter (eg, the outcome, such as birth defect, is already known in retrospective studies) [2]. Thus, the model followed by pregnancy exposure registries has distinct advantages over other study designs, because these registries can produce human data regarding medication safety in pregnancy while avoiding the ethical and logistical pitfalls of randomized controlled trials [5].

Despite the advantages over other study designs, pregnancy exposure registries face a number of challenges. Enrollment or recruitment is perhaps the most crucial issue, with most registries only capable of enrolling a small fraction of the exposed pregnancies, resulting in lack of power to assess specific malformations or health outcomes [6]. There may also be bias in the voluntary enrollment process [7], as women who agree to sign up to registries may already be aware of certain health conditions. Additional challenges include large dropout or lost-to follow-up rates [7], which result in the loss of information from many exposed pregnancies, the lack of availability of information before the discovery of the pregnancy, and incomplete reporting [8]. These challenges associated with pregnancy registries necessitate the exploration of additional sources of information for assessing drug safety during pregnancy.

### Motivation, Goals, and Contributions

Social networks have seen an unprecedented growth in terms of users worldwide. According to the Pew Research Report [9], nearly half of all adults worldwide and two-thirds of all American adults (65%) use social media, including 35% of those aged 65 years and older and over 90% of those aged between 18 and 29 years. Public health monitoring and surveillance research studies are therefore rapidly embracing the data made available through social media and developing tools that can effectively mine social media data [10]. Due to the limited amount of information that is available about pregnant women during premarket clinical trials and the challenges and disadvantages of existing prospective and retrospective surveillance approaches, there is a need to explore additional resources of information. Social media has the potential for serving as a crucial complementary resource for obtaining critical medication safety information following the release of medications into the market. The usability of generic social media for this task, however, depends on the successful development of systems that can actively identify pregnant women and collect relevant pregnancy-related data about them. This need is the primary motivation for the study reported in this paper. The specific goals of this paper were as follows:

- Design and validate a set of query patterns that can be used to retrieve posts that are highly indicative of pregnancy from Twitter users.
- Develop and evaluate a supervised machine learning approach that can accurately distinguish between real pregnancy-indicating tweets (PITs) and false positives.
- Design an end-to-end pipeline for collecting longitudinal data from the identified pregnancy cohort.
- Perform preliminary analyses of the extracted health timelines to assess their usefulness, identify limitations, and establish future research goals.

The main contributions of the paper are as follows:

- We present a mechanism and a set of queries by which large numbers of potentially pregnant women may be identified over social media.
- We present a supervised text classification approach for accurately detecting and enrolling a pregnancy cohort for data collection.
- We discuss a pipeline that incorporates the two aforementioned techniques to actively collect information posted by the detected pregnancy cohort.
- We discuss potential uses of the data collected from the cohort.

## Methods

### Preliminary Analysis

To assess whether social media can be utilized to identify cohorts of pregnant women, we performed a preliminary analysis using Twitter [11]. For the analysis, we employed one manually created query pattern of the form— “I.*(m|am|’m). *(weeks|months).*pregnant.” Tweets retrieved by the query were manually analyzed and grouped into two categories: PIT and not PIT. In total, 1200 retrieved tweets were labeled in this way, and 753 (62.75%) tweets were tagged as true PITs, whereas 447 (37.25%) were classified as false positives. This early analysis was very promising as it showed that tweets retrieved by such queries were quite likely to be real indications of pregnancy posted by the women themselves. In addition, the pattern collected over 1500 announcements per month, which suggested that in the long run, large cohorts could potentially be detected, particularly with the addition of new queries.

**Figure 1:**
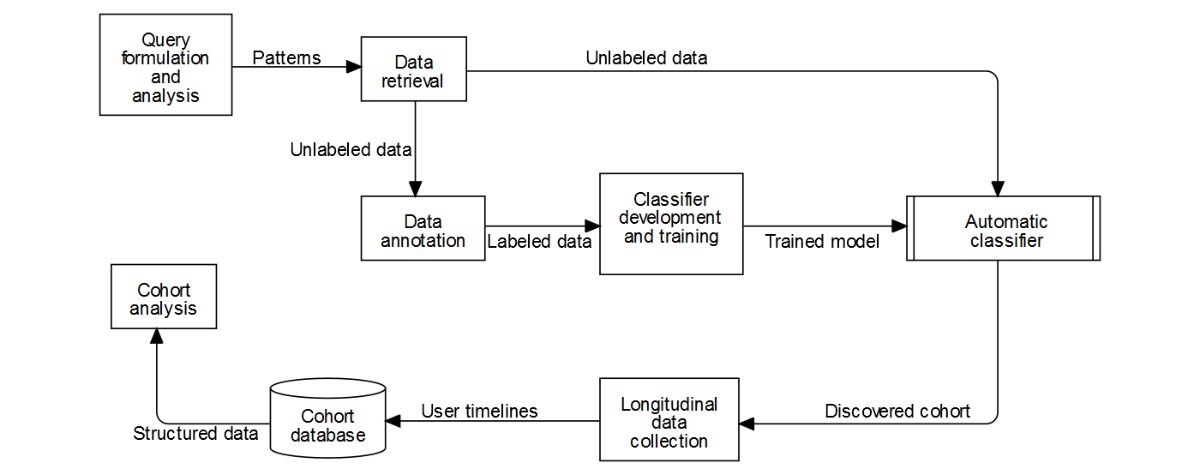
Flowchart for the pregnancy cohort discovery pipeline from social media.

In the same analysis, we also assessed the possibility of employing an automated supervised classifier to further filter the collected tweets so that pregnancy cohorts could be identified with greater precision. We experimented with several supervised classification approaches including Naïve Bayes and support vector machines (SVMs), and found the latter to produce acceptable performance with an F_1_ score of 0.80 (precision approximately 0.83) for the PIT class. These outcomes from our feasibility analysis study provided strong encouragement for us to further explore the problem and develop a more robust solution for cohort collection. We discuss the expansion of this preliminary study in the following subsections. Figure 1 presents a flowchart illustrating the overall workflow, beginning from the query formulation part until cohort analysis using structured data.

### Query Formulation

We manually studied the tweets identified by the preliminary study, and using the Twitter graphical interface (ie, the actual website), we identified additional high-frequency word n-gram patterns and rules by which PITs could be detected with high precision. For each potential pattern, we assessed its usefulness by manually using it as a query on the Twitter graphical interface. For each query, approximately 50 tweets were manually assessed. Patterns capable of retrieving approximately more than 60% true pregnancy posts were selected for large-scale retrieval. Patterns that retrieved large numbers of true positives, but with too many noisy false positives, were discarded, as we were primarily focused in ensuring high precision.

In this fashion, we identified 13 query patterns in addition to the pattern employed in the preliminary analysis. Once each query was identified, it was used to collect tweets from the Twitter Streaming application programming interface (API). This API exposes a sample of all the public tweets at real time and enables collection. However, the API does not allow the direct use of regular expressions. Therefore, we used the seed terms “pregnancy,” “pregnant,” “baby,” “family,” and “mom” to retrieve tweets from the API and then matched them with the specific regular expressions. The data collection module was run over a period of 18 months, with minor variations for each of the 14 queries. Table 1 presents the queries used along with estimates of the relative frequencies of tweets retrieved by them within a defined period.

### Annotation

A sample of the data gathered early on during the collection period was prepared for annotation. We observed early during the collection phase that there was a large variation in the number of tweets that were retrieved by each of the queries (as the third column in Table 1 indicates). To ensure that the distribution of the tweets in the annotation set represented the full set of retrieved data, we selected a stratified random sample of 14,300 tweets. An annotation guideline was prepared to ensure consistency in the annotation process. The annotation guideline is available in Multimedia Appendix 1.

Three annotators annotated all the tweets in a binary fashion, with overlapping annotations for 1000 tweets. Majority voting was used to resolve disagreement for the overlapping tweets. The interannotator agreement for the sample was κ=.79 (Fleiss kappa), which represents significant agreement. In total, 9819 tweets were tagged as true PITs, and 4338 tweets were annotated as false positives. These annotated tweets were then passed on to the next phase for training and optimizing an automated supervised classifier. Table 2 shows a sample of annotated tweets, with usernames deidentified.

### Classification

Out of the 14,300 completed annotations, 14,156 tweets were suitable for use in classification. The rest were removed for various reasons such as encoding issues and presence of another language. We explored a number of feature sets for effectively performing the classification task, including those that we had determined to be useful for social media text classification via our extensive past work in the domain [12-14]. In addition, we experimented with several popular supervised classification approaches to identify the best performing one on the problem, along with a baseline classification system. The classifiers we explored were SVMs, random forest (RF), and convolutional deep neural networks (DNN) with 3 hidden layers, and the baseline was Naïve Bayes. We divided the annotated dataset into an approximately 80-20 split (80% for training and system development and 20% for evaluation). We used the larger split for optimizing the classifiers and for identifying useful features. In line with our past research, we have made samples of the training data and additional resources publicly available for the research community [15]. To maintain a balance between privacy and reproducibility and to comply with Twitter’s data sharing policy, we will only share the tweets using their IDs, rather than the verbatim text. Therefore, all tweets deleted by the original posters will not be available to the public. The following is a description of the features we chose for our final classification system.

**Table 1:** Query patterns used for retrieving the pregnancy-indicating tweets and some notes specifying additional details. “.*” represents sequences of characters of any length, “|” represents “or” and “&” represents “and” in any order. Queries are shown in simplified forms. Frequency and relative frequency of tweets for each pattern is also shown (N=14,156).

Query pattern	Notes	Relative frequency, n (%)
(im|i am|i’m).*[*time*].* pregnant	Time can be week, weeks, month or months	4374 (30.90)
baby & arriving	N/A^a^	375 (2.65)
baby coming soon	Exact sequence with whitespace or punctuations in between	297 (2.10)
been.*[*time*] & since & i & pregnant	Time can be day, days, week, weeks, month or months; exact sequence for “been time” with whitespace or punctuations in between	22 (<1.00)
growing & baby & belly	N/A	150 (1.06)
(im|i am|i’m) expecting.*baby	Exact sequence for “(im|i am|i'm) expecting” with whitespace or punctuations. “baby” must appear anywhere after	74 (<1.00)
(im|i am|i’m) going to (b|be) a mom	Exact sequence with punctuations or whitespace in between	179 (1.26)
(im|i am|i’m) having a baby	N/A	1396 (9.86)
i (hav|have) been pregnant	N/A	88 (<1.00)
(ive|i’ve) been pregnant	N/A	735 (5.19)
adding & one & “our family”	Exact sequence for “our family” with punctuations or whitespace in between	13 (<1.00)
my pregnancy	Exact sequence with whitespace or punctuations in between	6211 (43.88)
(im|i am|i’m) going to have a baby	N/A	234 (1.65)
our family.*growing.*(2|two) feet	N/A	8 (<1.00)

^a^N/A: not applicable.

#### Word n-Grams

In text classification, word n-grams are typically the most informative features. These n-grams are preprocessed sequences of words, and they are excellent in capturing the meanings of text segments. We preprocessed the tweets by lowercasing them and performing stemming using the Porter stemming algorithm [16]. We used 1-, 2-, and 3-grams as features without the removal of stopwords, and during training, each tweet was represented as a vector of the counts of all the n-grams in the training vocabulary. In our preliminary study, we had also experimented with synonyms of certain terms, but we removed them from the final system as they did not appear to improve performance.

#### Dense Word Embeddings

A potential problem with n-grams, particularly with Twitter data, is that there may be a lot of variation within the set of n-grams, giving rise to very sparse vectors. Recently, the use of dense word vectors, or embeddings, has become popular in natural language processing (NLP) research [17]. These embeddings are learned from large volumes of unlabeled data, and they are capable of capturing semantic information about each word in the form of dense vectors. For this classification task, we obtained dense vector representations of each tweet simply by adding dense representations of all individual tokens. To obtain dense vector representations of the terms, we used publicly available pretrained vectors [18]. The vectors were learned from 400 million tweets, and each word was represented using a dense vector of size 400.

#### Word Clusters

One strategy to address the problem of sparse vectors in classification is to use generalized representations of terms that are created based on some predefined grouping criteria. In past work, we discovered that using cluster representations of words improves classification performance [19]. In our work, we used the Twitter word clusters provided by Owoputi et al [20]. These clusters are generated by first learning word embeddings from over 56 million tweets and then employing a hidden Markov model to partition the words into a hierarchical set of 1000 base clusters.

When generating features, we used the cluster number for each token in a tweet (if available) and represented the clusters as binary vectors. Therefore, the cluster vector for each tweet represented the general categories of words present in the tweets.

**Table 2:** Sample tweets retrieved by the 14 queries and their binary annotations. "True" indicates real pregnancy indications and "False" indicates false positives. For the true category, we have included at least one sample from each of the 14 queries.

Tweet	Category
one month today (give or take) I am going to be a mom...I can not wait to see what my baby girl looks like :-)	True
So I thought I would let Twitter know that I am expecting a baby in eight months!!!	True
this belly and the sweet baby growing inside is the best christmas gift I could ever ask for!!! Merry Christmas e...	True
been 3 weeks since I've heard bebes heart or seen it. So sometimes I don't feel pregnant but this new stretch mark is proving otherwise	True
Just s few short months from adding another one to our family!	True
Ready for Christmas and pumped to announce that baby boy **** will be arriving May 2017! #MC3	True
Pregnancy announcement Our family is growing by 2 feet and 1 heart	True
Hoping & praying for a solution to income issues. Baby coming soon! Need better #job & better #pay	True
i literally cannot wrap my head around the fact that I am going to have a baby in 16 days or less..	True
so I am having a baby and super excited	True
swear since I have been pregnant everyone's forgot about me and doesn't involve me in anything	True
well... im currently 39 weeks and 6 days pregnant... you can come any time now sweetie	True
i just took my pregnancy cravings to a whole new level: I put ranch on my macaroni and cheese. #Yummmmmmmm	True
i'm so crafty since I've been pregnant before I couldn't even color a rainbow.	True
forever amazed at the number of women that ask me when I am going to have a baby instead of asking me about my career goals.	False
i swear I've been pregnant for 2 years now. #theobesityneedstostop #ineedwine	False
I'm having a baby JB day and it's killing me. I love him so much @justinbieber	False
my sister is five weeks and three days pregnant. I’m going to be an auntie oh my god	False
girls will be two days pregnant already posting pictures talking bout “I’m getting big.”	False
Cant believe im having a baby brother!	False

#### Sentiment Features

Our inspections of the collected tweets during the preliminary analysis suggested that users might express strong sentiments when announcing their pregnancies, as can be seen in some of the examples from Table 1. Sentiment analysis itself is an active research area, and there has been a flurry of work in this domain, particularly for social media texts [21]. To capture the sentiments in the posts as features, we added features that represent sentiments in chosen scales. To each tweet, we assigned three sets of scores representing three different measures of sentiment based on the following: (1) lists of positive and negative terms [22], (2) prior polarities of terms [23], and (3) the subjectivity of the terms, which present both polarity and subjectivity [24].

#### Structural Features

These include features that present structural information about each tweet. The features include tweet length (in words and characters), number of sentences within the tweet, average lengths of sentences, and so on.

#### Experiments

For each of the four classifiers mentioned previously, we used the training set to explore features and identify near-optimal settings for specific hyperparameters, when appropriate, via 10-fold cross validation. The training set consisted of 11,325 tweets, and the test set consisted of 2832 tweets. These optimal settings for the classifiers were used to classify the tweets in the test set. In addition, we also combined the three classifiers to form an ensemble and predicted the test set labels via majority voting. The best performing classifier was then used to classify all the pregnancy-related tweets collected by our patterns. The entire annotated dataset was used for training before classification of the collected unlabeled data.

We also assessed the performance of the best classifier for each type of query pattern to understand whether tweets retrieved by specific queries require more attention. In addition, we performed an analysis of the learning rate of the classifier by performing classifications on the same test set with different proportions of the training set for training—starting at 1133/11,325 (10.0%) tweets and increasing by 10% at each step. We analyzed the receiver operating characteristic (ROC) curve at each training set size and also the overall performance to assess whether further annotation is likely to improve performance. We present the results in the next section, along with details about the contribution of each feature set. We used the python scikit-learn library for the SVMs and RF implementations and TensorFlow for the DNN implementation.

### Cohort Information Retrieval and Storage

All the user handles associated with the tweets classified to be positive by our chosen classifier were collected and stored. For each user, the Twitter Search API was used to collect all available past posts by the user, as per the restrictions of the API. In addition, new tweets posted by each of these users were collected on a weekly basis, resulting in the formation of a *timeline* for each user that encapsulates longitudinal information. All the information was stored in a Mongo database for future analysis.

A wide range of longitudinal information became available about each user’s pregnancy from the timeline. These included, but were not limited to, information about their medication usage, health habits (eg, smoking or drinking), and birth outcomes. Our detection and collection approach was targeted toward the large-scale analysis of this information. We present some of the possibilities in the Discussion section and leave the specific analyses for future work, as that is beyond the scope of this study.

### Health Information Analysis

We performed several preliminary level analyses using the collected data to assess the utilities of the timelines, their potential use in future studies, and the NLP-oriented future work required to increase their usefulness. These analyses included the following: (1) assessing the possibility of detecting trimester information from the collected cohort, (2) determining the presence of medication-related information for the cohort members, and (3) determining the presence of information regarding miscellaneous health conditions in the timelines. We now briefly discuss these analytical methods.

#### Trimester Detection

The duration of a pregnancy may be divided into three trimesters: first—week 1 to week 12, second—week 13 to week 27, and third—week 28 to birth. Trimester information is crucial for the future analysis of the pregnancy cohort as health events (eg, medication intake) may affect the fetal outcome uniquely, depending on the trimester. To successfully identify the trimester associated with a posted health-related event, information about the pregnancy start date is required. Our analysis of a sample of timelines suggested that the key NLP challenge in this problem is to detect the statements regarding the progress of the pregnancies, which are often available in the pregnancy tweets retrieved by our queries. We employed a simple, rule-based approach to assess the portion of the pregnancy cohort from which trimester information could be derived. In our rule-based algorithm, we first attempted to identify all tweets within a timeline that mentioned the terms “pregnant” and “pregnancy” (seed word). Next, terms occurring within a symmetric context window of size 6 of the seed term were collected. Within the context window, the algorithm then searched for key temporal terms such as “week“ and “month,” along with the presence of a number mention (eg, “6,” “12,” “18,” and so on). If all these rules were satisfied, the number mention and the temporal term mention were used to determine the progress of the pregnancy (eg, “6,” “week,” and “pregnancy” in "6 weeks into the pregnancy"). The number and the other mentioned terms were extracted and compared with the time stamp of the associated tweet to identify the approximate start date and trimester of the pregnancy.

#### Medication Mention Analysis

Medication intakes during pregnancy and their potential links to fetal outcomes is an important research topic, as discussed earlier in the paper. Pregnancy registries are currently the only source of information regarding this. In the future, if social media is to be used as a complementary source for studying medication safety during pregnancy, there must be intake-related information available within the collected pregnancy timelines. Although a full study is outside the scope of this paper, we performed a preliminary assessment by automatically computing the frequencies of mentions of a set of medications on a sample of our data (the same sample for which potential trimester information was detected). The goal was to ascertain whether medication usage information is available, rather than to perform a thorough analysis, which we leave as future work.

#### Assessment of Availability of Health Conditions

We manually analyzed a small sample of 30 user timelines to identify the types of health information that were present and also to ascertain what future tasks are necessary to improve the utility of the collected information. We present a sample timeline in the Results section and provide further details in the Discussion section.

## Results

### Classification Results

The final training set consists of 7830 instances of the pregnancy class and 3494 instances of the nonpregnancy class. The test set consists of 1989 instances of the pregnancy class and 843 instances of the nonpregnancy class. Table 3 presents the performance of the classifier on the test set. From the table, it can be seen that the three nonbaseline classifiers and the ensemble perform similarly in terms of pregnancy class F_1_ score. The performances of the SVMs and DNN are better than that of the RF classifier, although these performances are not statistically significant. The ensemble of the three classifiers performs marginally better than the others on the test set, but the improvement is not significant and comes at a very high price in terms of time (eg, it is approximately 5 times slower to run than the stand-alone SVMs). All these classifiers significantly outperform the Naïve Bayes baseline.

On the basis of these results on the annotated set, we chose to use the SVMs in our system. Compared with the DNN, the SVMs appear to have marginally higher precision, which is preferred in our overall pipeline. Note that there is a possibility that using deeper DNNs would result in better performance, as typically is the case. However, deeper networks would also be computationally much more expensive, and so we did not include them in our exploration. SVMs performed much faster than both the DNN and the ensemble. Thus, consideration of all these factors favored the use of the SVMs.

**Figure 2:**
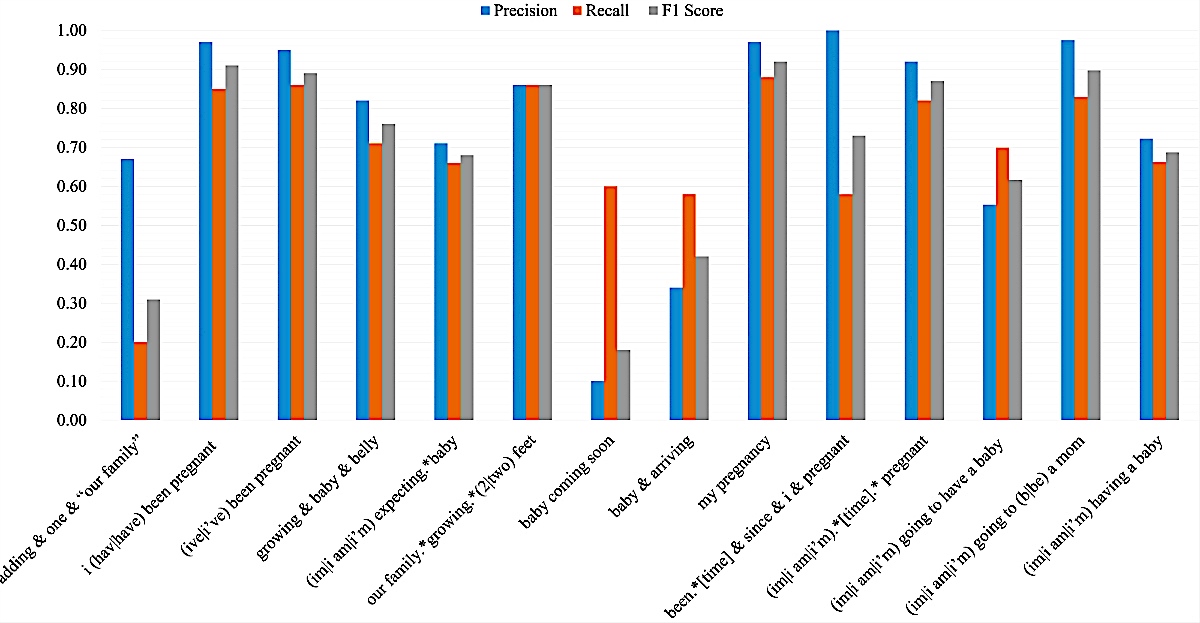
The performance of the supervised classifier on the pregnancy class for each query pattern.

Figure 2 presents the performance of the chosen classifier on posts retrieved by each of the query patterns. These performance results were obtained via 10-fold cross validation over the entire annotated set. The figure shows that for the two queries with the largest retrieval rates ("(im|i am|i'm).*[time].*pregnant" and "my pregnancy"), the performance scores were better than the overall averages. This is likely because of the fact that the annotations were carried out on a stratified random set, and therefore, the total number of annotated tweets for these sets was much higher than that for others, leading to the better training of the algorithms for these patterns. The pattern “(im|i am|i’m) having a baby” has the third highest retrieval rate, but the performance of the classifier is much lower for this set, which drives the overall performance down. In general, the patterns with low retrieval rates appear to perform poorly from the figure. We provide a brief analysis of the causes of errors in the Discussion section.

**Figure 3:**
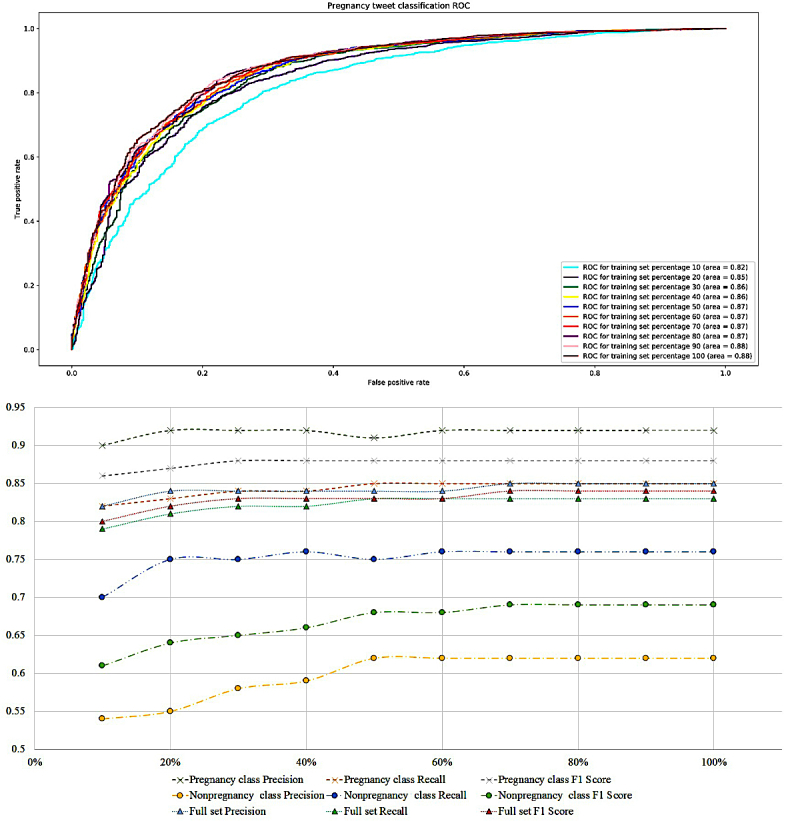
Receiver operating characteristic (ROC) curves for the pregnancy tweet classifier at different sizes of the training data (top). Values for the area under the ROC curve (AUC) are also shown for each training set proportion. Classification precision, recall and F1 score over each class and the full dataset at each training set proportion (bottom).

Figure 3 provides further insight into the performance of the system. The ROC curves in the figure (top) show that once over 50% of the training data are used, the prediction performances remain fairly stable. This suggests that further annotations of the same type of data are not likely to improve performance of the classifier. The learning rate chart (bottom) shows the performance metrics over the two classes and the full dataset at different sizes of the training data. This chart also shows that for each set, the performances remain stable after about 60% of the training set size. Unsurprisingly, as the training set size is increased, the biggest improvements are seen in the performance metrics of the smaller nonpregnancy class. As the performance over this class improves, so does the overall performance, albeit marginally.

Table 4 presents the performances obtained by the classifier during leave-one-out and single feature experiments. Recall, precision, and F_1_ score for each class and the full set are shown. In none of the leave-one-out experiments, the performance of the combination of features drops significantly when a single feature is removed. The removal of n-grams results in the largest drop, but it is only marginal. This suggests that the performance of the classifier is not dependent on any of the single features but on the combination of all the features. This is desirable in a classifier for Twitter data because the low number of words in each tweet means that one type of feature may often not be able to capture enough information to perform classification correctly. Incorporating a number of features increases the chances of correct classification. The single feature scores in the table give a clearer idea of which features are most informative when employed in a stand-alone manner. Unsurprisingly, n-grams appear to be the strongest set of features and result in performances that are very close to the best performance of the classifier. Dense vectors and word clusters also produce good performances on their own, verifying the usefulness of these two feature sets. Structural features and sentiment features, although proved to be useful in our preliminary study using a much smaller training data, do not contribute significantly once the training set size is sufficiently increased. For these two features, large drops in performances are observed when they are used stand-alone. In all cases, we see a greater drop in the nonpregnancy class compared with the pregnancy class once a feature or a combination of features is removed. Although our focus is the pregnancy class, it is crucial to improve performance over the nonpregnancy class as changes in performance in one class directly affect the performance in the other.

### Cohort Collection Statistics

Over a period of 18 months, the data collection component of our system (retrieval and classification) collected a total of 71,954 potentially pregnant users. Past data collection of the users resulted in the collection of over 250 million tweets, at about 3500 tweets per user on average. New pregnant users were detected at a rate of approximately 9000 to 10,000 per month, and 25 to 35 million new tweets were detected on average during the same period. At this rate, we expect the collection of an additional 100,000 to 120,000 timelines in the next 12 months.

**Table 3:** Classifier performances for the three strong classifiers, the Naïve Bayes baseline, and the ensemble classifier. Precision, recall, and F_1_ score for the pregnancy class for each classifier are shown along with overall accuracy and 95% CI for the accuracy.

Classifier	Pregnancy class			Both classes
	Precision	Recall	F_1_ score	Accuracy (95% CI)
Naïve Bayes	0.44	0.90	0.59	0.57 (0.56-0.58)
Random forest	0.95	0.79	0.86	0.81 (0.80-0.82)
Deep neural network	0.90	0.87	0.88	0.84 (0.83-0.85)
Support vector machines	0.92	0.85	0.88	0.84 (0.83-0.85)
Ensemble	0.93	0.85	0.89	0.84 (0.83-0.85)

**Table 4:** Leave-one-out and single feature scores for the features used in classification. “-” indicates that the feature was removed.

Feature set		Pregnancy class			Nonpregnancy class			Full set		
		P^a^	R^b^	F^c^	P	R	F	P	R	F
**All**		0.92	0.85	0.88	0.62	0.76	0.69	0.85	0.83	0.84
	-N-grams	0.90	0.85	0.87	0.61	0.73	0.67	0.83	0.82	0.82
	-Dense vectors	0.92	0.85	0.88	0.61	0.76	0.68	0.84	0.83	0.83
	-Word clusters	0.92	0.84	0.88	0.58	0.76	0.66	0.84	0.82	0.83
	-Sentiment features	0.92	0.85	0.88	0.62	0.76	0.68	0.85	0.83	0.84
	-Structural features	0.92	0.85	0.88	0.62	0.76	0.68	0.85	0.83	0.84
N-grams		0.92	0.83	0.86	0.55	0.76	0.63	0.84	0.81	0.82
Dense vectors		0.89	0.82	0.85	0.54	0.68	0.61	0.81	0.79	0.80
Word clusters		0.90	0.83	0.86	0.56	0.70	0.82	0.82	0.80	0.81
Sentiment features		0.70	0.64	0.67	0.28	0.20	0.24	0.55	0.49	0.52
Structural features		0.67	0.69	0.68	0.30	0.28	0.29	0.55	0.56	0.56

^a^P: precision.

^b^R: recall.

^c^F: F_1_ score.

### Health Information Analysis Results

We applied our pregnancy trimester extraction algorithm on 34,895 user timelines who were classified to be pregnant by our classifier in the early part of the study. Our algorithm detected pregnancy trimester information for 15,523 (approximately 44%) users. The algorithm further categorized each tweet belonging to these timelines into one of the three trimesters. Although detection of the availability of trimester information was highly accurate, manual analysis of a small sample of the timelines suggested that the algorithm was accurate in only about 50% of the cases in terms of categorizing the timelines into trimesters. This verified that trimester information is available in a large sample of our cohort, but a more robust algorithm is required for automatic categorization of information into trimesters.

**Figure 4:**
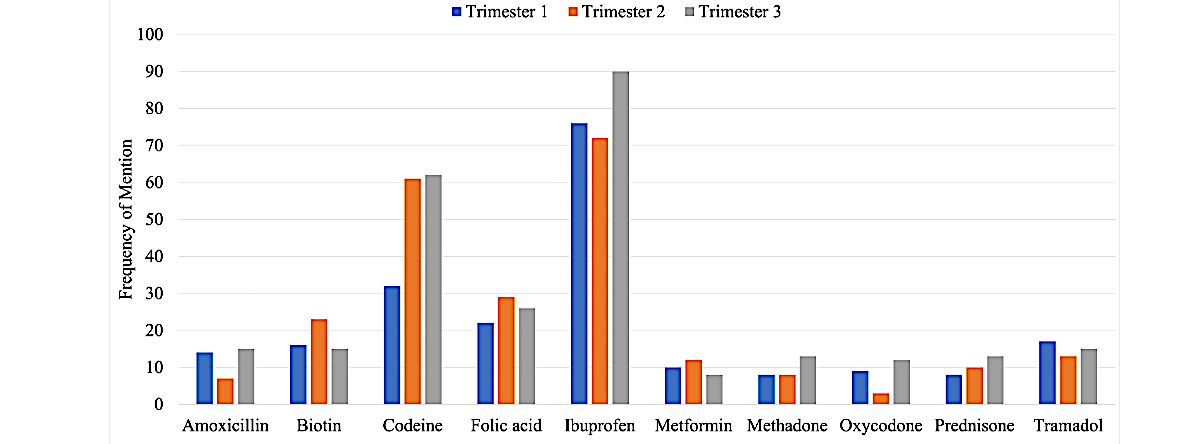
Distribution of mentions of a set of medications in the data collected for a sample of our collected pregnancy cohort. Mentions are also categorized by our preliminary trimester detection approach.

Computation of medication mention frequencies on the same sample for which trimester information was detected verified that there is some medication-related chatter available in the timelines. Figure 4 shows the distribution of popular drug mentions across the pregnancy trimesters for Twitter users. Manual analysis of the previously mentioned timelines, however, showed that only a sample of the medication mentions are real examples of intake. In addition to medication mentions, the analysis revealed that a variety of other health-related information could potentially be mined from the timelines. This information, however, is intertwined with a large amount of noise. Table 5 shows sample posts in chronological order from one of the timelines that we manually analyzed, illustrating some of the types of information that are available. From Table 5 it can be seen that tweets 8, 11, and 15 present information regarding the progress of the pregnancy, and paired with the time stamps on the tweets, this information can be used to identify the trimester of a post.

**Table 5:** Sample of 20 relevant posts from the timeline of a user classified as pregnant by our system. The posts were manually curated and categorized. Usernames have been anonymized.

Number#	Tweet	Trimester	Information type or comment
1	The tonsils are being evicted...1st October they will be no more!	First	Health condition
2	God...I’m 30...The right thing to do is probably to eat lots of cake to make it better #happybirthdaytome #30s	First	Age of user
3	@username yep I’m all done, not feeling too bad at the moment, got a pharmacy full on painkillers to see me through! Thanks xx	First	Medication
4	@username thanks, had some toast before I came home, just made a second batch to take painkillers with!	First	Medication
5	@username hazel on dw worked out the adult dose of calpol the other day so I saved that for emergencies, we always have calpol in! Xx	First	Medication
6	@username @username I’ve had tramadol this week post tonsils and can confirm that it definitely leaves you feeling pissed and sleepy!	First	Medication
7	poor you doesn’t sound fun. I’m ok, throat is much better, off all the painkillers now which is good!	First	Stopping medication
8	20 week scan today! So pleased its first thing and I don’t have to wait all day. Big question is, pink or blue??	Second	Progress information
9	So baby conn number 2 is a girl! Alex was right all along, now I need some nice girl names! #baby	Second	Gender of baby or health condition
10	Looking forward to #oneborneveryminute, love baby shows even if I am 25 weeks pregnant	Second	Pregnancy post detected by our query
11	Nothing like seeing a tiny baby to make me realise I’m getting one of those soon #10weekstogo #realitycheck	Third	Progress of pregnancy
12	Looks suspiciously like we are joining the pox bench, anyone else? #chickenpox	Third	Health condition
13	Think we may over the worst for the pox, day 5 no new spots but lots crusted over. #poxwatch	Third	Health condition
14	me! 36 weeks pregnant and travelling from sunny weston super mare to see you!	Third	Pregnancy indicating post
15	@username i am not burnt as I am mainly inside or in the air conditioned car where I am cooler #37weekspregnant	Third	Progress of pregnancy
16	This is Charlotte Amelia Conn born today at 11:22 weighing in at 7lb 10ozs	Birth	Birth announcement
17	@username Charlotte was 7lb 10ozs and dropped to 6lb 12ozs today. Apparently anything over a 10% drop triggers a whole load of stuff	Post birth	Weight loss in newborn
18	@username thought you’d think so! Yeah apparently 10% is the cut off, hers was an 11.2% drop. Hoping to avoid readmission tomorrow	Post birth	Weight loss in newborn
19	@username yup. She needed to gain and she lost another 10g. :-(	Post birth	Continuing weight loss
20	@username does indeed suck to be a grown up. We are good now thanks, Lottie’s gaining weight well too. Need a bit more sleep though!	Post birth	Newborn regaining weight

## Discussion

### Principal Findings

The goal of our study was to determine whether cohorts of pregnant women could be detected using publicly posted social media data and natural language processing. We designed queries for retrieval of user posts that strongly indicate that the user is pregnant. Following the collection of such posts, supervised classification was used to further filter out false positives and collect a set of users that were highly likely to be pregnant. The results obtained show that such an approach was capable of detecting pregnant women and creating a social media–based pregnancy cohort that can be used for further analysis. Our overarching goal is to complement existing sources of pregnancy safety information with data mined from social media data. To be able to do that, the first, and most crucial, step is to be able to detect pregnant women with high accuracy or precision. Our study confirmed that by using carefully constructed queries and a well-designed supervised classification strategy, this can be achieved. Although the queries were able to collect PITs with varying accuracies for each query, the supervised classification approach had an overall F_1_ score of 0.88 for the pregnancy class, which was almost equal to human agreement on these data. This suggests that our model was indeed effective in accurately detecting pregnancy cohorts from noisy social media data.

In addition to the performance of our classifier, the large volumes of user posts collected from the cohort and the health-related information detected via our small-scale analysis strongly support our initial motivation for such a system. The data collected from the cohorts appear to encapsulate, based on our small postclassification analyses, crucial knowledge regarding a variety of health-related information, albeit within an abundance of noisy, irrelevant information. The data may therefore be used for studying potential associations between medication intake and pregnancy outcomes, maternal health patterns, behavioral patterns and their associations with pregnancy outcomes, health of newborn children, and many more.

Our supervised classification approach also has the potential of being applied to other similar problems. Our approach combines sparse and dense vectors that independently perform well in the classification task. Such a combined representation is likely to benefit other social media text classification tasks that use short text nuggets, with limited contextual information, as input.

### Applications of Automated Cohort Detection

As mentioned in the first section of the manuscript, certain cohorts such as pregnant women are not included in clinical trials. Thus, drug safety information for pregnant populations is typically not known when medications are released into the market, and discovering new associations to adverse effects may take years. The development of successful monitoring techniques utilizing social media data may expedite the process of discovery of unknown associations. Such techniques will have to be developed on top of our detection mechanism in the future. In addition, social media may provide information about the mothers’ behavioral patterns, which they may not reveal to their doctors. Such patterns may include smoking, drinking, depressive behavior, and prescription medication abuse. Such information may help derive causal associations with adverse fetal outcomes and postpregnancy maternal health.

Our framework may be used to detect and monitor other cohorts as well. The key is to be able to identify queries that can retrieve posts where users *subscribe* to a cohort and an automated classification strategy that can filter out noise. The strategy can be used, for example, to detect users suffering from particular health conditions, users of a particular medication or medical intervention, and users addicted to prescription or illicit drugs.

### Error Analysis and Linguistic Analysis

#### Error Analysis

We performed a limited error analysis to determine what factors commonly caused errors. The results of the analysis were in strong agreement with the per query result break down shown in Figure 2. To summarize, we found large proportions of errors for three specific query patterns—“baby & arriving,” “baby coming soon,” and “adding.*one ‘our family’.” In the first two cases, the term “baby” was found to be often used to refer to a loved one rather than to refer to a to-be born child. Both these queries had decent representation in the total annotated data (>2%), but the many variations meant that it was not possible for an automated algorithm to distinguish real announcements from false positives. For the third query, we found that although some tweets genuinely indicated the birth of an upcoming child from the mother, others referred to unrelated life events such as getting a new pet or getting married. Some were also posted by other family members and not the pregnant woman and thus were considered to be false positives according to our guideline.

On the basis of these common error cases, we envision several possible solutions that can be attempted in the future to further improve classification accuracies. As we selected a stratified random sample for annotation, some of the tweets retrieved by the patterns with low retrieval rates only received a small number of annotations. Therefore, it is likely that performance over those tweets will improve if more of them are annotated. However, considering their low retrieval rates, it may be prudent to simply remove such error-prone patterns from our future retrieval effort. As for tweets from the male counterparts of pregnant women that are detected by our queries (eg, the query patterns that include *our*), a module can be added to our pipeline that attempts to automatically detect gender from user timelines. We will consider the development of such a module or component in the future.

#### Linguistic Analysis

Gaining insight into the linguistic features that characterize and differentiate the “pregnancy” and “nonpregnancy” tweets could inform modifications to the queries for future data retrieval. To gain such insight, we drew upon a tool for corpus analysis called *DocuScope* [25]. On the basis of DocuScope’s classification and frequency counts of linguistic patterns in 3000 of the “pregnancy” tweets and 3000 of the “nonpregnancy” tweets, we conducted a *factor analysis* [26] to explore the features that frequently co-occur in the tweets. Finally, we used the results of the factor analysis—in particular, the factor scores—as input for analysis of variance (ANOVA) to assess whether any of the factors (ie, groups of highly correlated linguistic features) explain significant linguistic variation [27] between the two groups of tweets.

One of the factors in the analysis reveals that the words *pregnancy* and *pregnant* frequently occur with first-person references (eg, *I* and *my*) in “pregnancy” tweets, whereas references to other people (eg, *she*, *he*, *brother*, and *sister*) and goal-oriented actions (eg, *having a baby*) are frequently absent in “pregnancy” tweets and vice versa for “nonpregnancy” tweets. The salient features in many of the “nonpregnancy” tweets aggregate to announce that a sibling is having a baby (eg, *my sister is having a baby*) or that the author of the tweet is going to be a sibling (eg, *I’m having a baby brother*), whereas the salient features in many of the “pregnancy” tweets combine to announce the author’s own pregnancy. According to ANOVA, this factor explains statistically significant linguistic variation between the two groups of tweets.

Factor analysis can shed light on the micro-level linguistic cues that latently contributed to the annotators’ high-level decisions to classify the tweets as “true” or “false” indications of pregnancy; consequently, it may also provide insight into the linguistic features that are playing an influential role in the automatic classification of the tweets. For instance, knowing that first-person references are a salient feature of “pregnancy” tweets might explain the relatively weaker performance of the classifier on the “having a baby” query pattern; in tweets such as “I’m having a baby brother,” the *I’m* might be confusing the classifier into thinking that this is a “true” pregnancy announcement. Such insight could inform modifications to the queries for future data retrieval.

### Limitations

This study has several methodological limitations that warrant further research. First, the cohort members for this study were chosen from a single social network, Twitter. Twitter is unique as a social media resource as posts can have a maximum length of 140 characters. This presents numerous problems to NLP tools because of lack of context, alternate spellings, and so on [28], but this property also limits the number of patterns that can be used to describe pregnancy-related information. Extending our framework beyond Twitter will require customizing queries to the social network chosen and the training of supervised learning algorithms with additional data.

The population reached through Twitter is also limited, and the sample is biased to social network users only. However, such biases exist in all samples for similar tasks, and social media is perhaps the most efficient way to reach, communicate, and collaborate with a large, diverse population [29,30]. A more important limitation of using social media is that complete information about individual cases may be harder to obtain, unlike traditional epidemiological studies. Although large numbers of cohorts can be detected, not all their health-related activities and health conditions may be available from their posts. The benefits of large cohort size may be diminished because of this. There is also the problem of discovering demographic information—only limited or no information regarding individual user demographics may be available. In some cases, the geographic locations of the users are available. Other demographic information such as age and race need to be determined via automated techniques. Reliable techniques for discovering demographic information for the pregnancy cohorts must be developed in the future. At this point, however, the use of social media in a manner that we have described appears to be very promising to complement traditional sources in the future.

### Comparison With Prior Work

To the best of our knowledge, there is currently no existing work that attempts to identify cohorts of pregnant women over social media for large-scale drug safety analysis. Social media–based research has primarily focused on more generic surveillance tasks such as influenza spread forecasting [31,32], pharmacovigilance [33], medication abuse monitoring [13,34], and drug-drug interaction [35] to name a few. Most of the social media–based studies attempt to derive conclusions from information at the post level, rather than attempting to derive associations from longitudinal data. Some studies have utilized simple detection methods to identify users with specific characteristics and then analyzed the posted information. Correia et al [35], for example, used hashtags on Instagram to collect user timelines and detect drug-drug interactions, and De Choudhury et al [36] utilized Twitter data to predict postpartum depression. Hoang et al [37] assessed the feasibility of detecting detrimental prescribing cascades from Twitter user timelines. However, as discussed by the authors, such detection is challenging because of uncertainty and rarity of social media data. The work presented in this paper goes beyond these prior works by establishing a thorough and accurate approach for detecting a specialized cohort and also provides a novel opportunity to perform safety surveillance for pregnant women using publicly available data.

### Conclusions

In this paper, we presented an approach for automatically identifying large cohorts of pregnant women over social media. Our proposed two-step approach for this detection first identifies potential pregnant women using targeted queries and then employs supervised classification to filter out most false positives. We thoroughly evaluated our cohort identification and classification approaches to validate that this is a viable approach for pregnancy cohort detection. We also showed potential uses of the information collected and future tasks.

On the basis of the findings of our study, social media promises to be a useful resource for performing drug safety research on pregnancy cohorts, particularly given the drawbacks associated with other sources including pregnancy registries. It must be noted, however, that social media is not expected to replace these traditional sources but rather serve as a complementary resource. An identical pipeline may also be used for automatic detection of other types of cohorts. Future research, with specific targeted applications of the data collected, will provide further insight regarding its usefulness.

## Appendix

Multimedia Appendix 1 Pregnancy tweet annotation guideline.

## Abbreviations

ANOVA: analysis of variance
API: application programming interface
DNN: deep neural network
NLP: natural language processing
PIT: pregnancy-indicating tweet
RF: random forest
ROC: receiver operating characteristic
SVM: support vector machine
