# Supplementary material for: Discovering Cohorts of Pregnant Women From Social Media for Safety Surveillance and Analysis
Source: J Med Internet Res. 2017 Oct 30;19(10):e361. doi: 10.2196/jmir.8164 (PMC5684515; doi:10.2196/jmir.8164)
Supplement: Multimedia Appendix 1 [file jmir_v19i10e361_app1.pdf]

Annotation Guidelines for Pregnancy Cohort Project

Version Tracking		
**Date**	**Changes and Comments**	**Author**
January 18, 2017	Initial guideline creation	Arjun Magge
January 20, 2017	Minor modifications	Abeed Sarker

Objective

The objective of this document is to define the annotation guideline for the binary annotation of tweets containing pregnancy related tweets from twitter. Individual tweets will be marked *positive* if there are one or more indications in the tweet that the person tweeting it is/was pregnant. It will be marked *negative* if no such indication was present.

The project focuses on identification of the cohort of pregnant women on twitter. Hence, legitimate pregnancy related tweets will be marked *positive* if ANY of the following are true:

1. The user states that she is pregnant (includes cases where the tweets do/don't have additional information about the phase of pregnancy in terms of weeks/months).
2. The user states that she was pregnant previously.
3. There is a reference in the tweet that the user is/was pregnant.

The tweets will be marked *negative* if ANY of the following are true:

1. The user is not female.
2. The tweet is entirely about someone else's pregnancy without a reference to their own.
3. The tweet is about taking a pregnancy test but has not evidence of a positive result.
4. The handle-name/tweet-contents indicates that the user is an organization/blog.
5. The tweet is about "looking pregnant" after having consumed food.
6. The tweet indicates the pregnancy phase is over 42 weeks or 9 months.
7. There is evidence that the tweet was a quote from a movie/tv.

Challenges and Precedence

When the text in the tweet is too short or the content is too vague, it might be difficult to make a decision regarding the class of the tweet. In such cases, the handle name can be used to aid the decision. When a tweet does not belong to any of the described qualifiers, the annotator can make a subjective assessment to mark the tweet as positive/negative.

As a rule of thumb, absence of evidence of the user being pregnant can be marked *negative*.

Annotation Target

The target is to have a total of 15,000 tweets annotated by 2-3 members

1. For two members, 1000 common tweets for kappa calculation and 6000 tweets per member.
2. For three members, 1000 common tweets for kappa calculation and 4000 tweets per member.
